# Perceptual Learning and Aging: Improved Performance for Low-Contrast Motion Discrimination

**DOI:** 10.3389/fpsyg.2013.00066

**Published:** 2013-02-20

**Authors:** Jeffrey D. Bower, Takeo Watanabe, George J. Andersen

**Affiliations:** ^1^Department of Psychology, University of CaliforniaRiverside, CA, USA; ^2^Department of Cognitive, Linguistic and Psychological Sciences, Brown UniversityProvidence, RI, USA

**Keywords:** perceptual learning, aging, motion perception, spatial suppression, contrast

## Abstract

Previous research has shown age-related differences in discriminating motion at different levels of contrast (Betts et al., 2005, 2009, 2012). A surprising result of this research is that older as compared to younger observers showed improved performance in detecting motion of large high-contrast stimuli suggesting age-related differences in center-surround antagonism. In the present study we examined whether perceptual learning methods could be used to improve motion discrimination performance for older individuals under high- and low-contrast conditions. The stimuli were centrally presented Gaussian filtered sine-wave gratings (Gabors) that were either 5° or 0.7° diameter with contrast of 0.92, 0.22, or 0.028. Older and younger participants received 3 days of training. The task was to identify if the motion direction was leftward or rightward. Duration thresholds for motion discrimination were derived using two randomly interleaved staircases and compared between pre-/post-test sessions. Both older and younger subjects showed lower duration thresholds as a result of training. The improved performance, for older subjects, due to training was observed for all size and contrast conditions, with training with small low-contrast stimuli resulting in a 23% improvement in motion discrimination performance. Older observers, as compared to younger observers, did show evidence of decreased spatial suppression across all contrast levels. These results suggest that perceptual learning techniques are effective for improving motion discrimination performance, especially for conditions that are difficult for older individuals.

## Introduction

It is well documented that many aspects of visual processing decline with advanced age. Previous research has shown that age-related declines in processing orientation, luminance, contrast, and motion are not the result of changes in optics due to the aging eye (see Owsley, 2011; Andersen, 2012 for reviews). In addition, declines have been observed for mid- and high-level aspects of visual processing such as form, depth, slant, and 3D shape perception (see Andersen, 2012). Age-related declines in motion processing have been the focus of a considerable amount of research (see Hutchinson et al., 2012 for a review), and has been examined using several different types of motion stimuli – drifting sine-wave gratings, coherent motion random dot cinematograms (RDCs), and global motion RDCs. Early research on motion and aging found decreased sensitivity for older as compared to younger observers for drifting sinusoidal gratings, particularly low frequency gratings (Sekuler et al., 1980). More recent research (Snowden and Kavanagh, 2006) found age-related declines over a wide range of spatial frequencies. Studies examining coherent motion RDCs (the perception of the direction of motion of moving dots imbedded in noise) found significantly higher thresholds with increased age, with thresholds for individuals over age 70 twice that of younger college age individuals (Trick and Silverman, 1991). This finding has been replicated in several studies (Gilmore et al., 1992; Andersen and Atchley, 1995; Snowden and Kavanagh, 2006; Billino et al., 2008) including research on motion sensitivity and retinal eccentricity (Atchley and Andersen, 1998). Finally studies examining global motion RDCs (dots that move in an average motion direction with each dot motion path containing a random motion component) found clear evidence of age-related declines in the detection and identification of motion direction (Bennett et al., 2007) and proposed a model suggesting that age-related declines were due to the bandwidth of individual motion channels.

Previous neurophysiological studies (Schmolesky et al., 2000; Hua et al., 2006; Yang et al., 2008) have suggested that changes in visual processing due to aging are the result of declines in inhibition, possibly due to declines in levels of γ-aminobutyric acid or GABA. Psychophysical studies have also suggested that declines in visual processing with age, such as reduced motion perception, are the result of declines in inhibition. Evidence in support of age-related changes in inhibition and motion processing has been observed in a psychophysical study examining motion discrimination with high- and low-contrast stimuli (Betts et al., 2005, 2009, 2012). Subjects were presented with drifting Gabor patches that varied in size and contrast and asked to discriminate if the motion direction was leftward or rightward. Duration thresholds were derived and indicated the minimum amount of time needed to perform the task accurately. Younger observers had elevated thresholds with high-contrast (92%) and large (5°) stimuli – a result consistent with previous research (Tadin et al., 2003) suggesting that elevated thresholds for large-size high-contrast stimuli, as compared to small-size high-contrast stimuli, were due to center-surround antagonism. In Betts et al. (2005), both younger and older observers’ thresholds also increased with increased contrast for large stimuli. However, older observers had lower duration thresholds than younger observers for high-contrast large stimuli. These results suggest that reduced center-surround suppression – possibly due to decreased neural inhibition – occurs with increased age (Betts et al., 2005).

Given the extensive evidence of age-related declines in motion processing an important question is whether any methods can be used to improve visual function. One approach would be to use training protocols found to be effective in studies on perceptual learning (PL; see Fahle and Poggio, 2002; Fine and Jacobs, 2002; Sagi, 2011). Recent research has found that perceptual learning protocols are effective for improving vision among older individuals. These studies have shown that PL can be used to improve texture discrimination (Andersen et al., 2010) and motion processing (Bower and Andersen, 2012). Bower and Andersen (2012) used the perceptual template model (Lu and Dosher, 1999, 2008; Lu et al., 2006) to assess age-related differences in motion processing by comparing perceptual efficiency between age groups both before and after perceptual training. The perceptual template model assumes that human perceptual performance is limited by inefficiencies in visual processing and that perceptual learning is a result of increased perceptual efficiency due to reduced internal noise and/or increased tolerance to external noise. Bower and Andersen (2012) used the model to examine age-related changes in additive internal noise and tolerance to external noise as a result of training. The results indicated elevated internal noise for older as compared to younger observers prior to training – a finding that is possibility related to the results suggesting reduced inhibition for older individuals in motion processing (Betts et al., 2005). In Bower and Andersen (2012), PL training resulted in decreased internal noise for older individuals. If changes in internal noise are related to changes in neural inhibition then reduced internal noise resulting from PL training might increase inhibition resulting in a change in center-surround inhibition and thus alter motion thresholds.

The purpose of the present study was to examine whether the use of PL protocols would result in changes in motion processing for older individuals due to changes in center-surround inhibition. To examine this issue we used stimuli that were matched to a subset of conditions examined by Betts et al. (2005), in which observers were presented with drifting Gabor patches that varied in contrast and size. Specifically, Betts et al. (2005) used Gabor patches at seven contrast levels (92, 46, 22, 11, 5.5, 4.2, and 2.8%) and four sizes (5.0°, 2.7°, 1.3°, and 0.7°) and found that older observers performed better than younger observers in the largest size tested (5.0°) at contrasts above 22%. In the present study we used two sizes (5.0° and 0.7°) and three contrasts levels (92, 22, and 2.8%) for a total of six unique conditions. These conditions represent the extreme size and contrast levels of the study by Betts et al. (2005) and include the conditions that resulted in age-related differences in center-surround inhibition.

The present study was run over a 5-day period, and included a pre- training and post-training assessment on separate days with three intervening training days. If the use of PL protocols results in increased inhibition for older observers then we should observe a reversal of the improved performance for older observers over younger observers with high-contrast large-size stimuli.

## Materials and Methods

### Participants

The participants were nine younger (mean age 21.0) and nine older (mean age 66.8) observers (see Table 1 for additional demographic information). The younger participants were recruited from the undergraduate population at the University of California, Riverside. The older participants were volunteers from continuing education courses at the University of California, Riverside’s Extension center and volunteers obtained through a direct mailing for subject recruitment in the Riverside community. All participants were paid 10 dollars per hour of experimental time plus an additional bonus of 25 dollars after completing the last day of the experiment. All participants had normal or corrected to normal vision.

**Table 1:** **Means and standard deviations of participants’ demographic information and results from perceptual and cognitive tests**.

Variable	Younger		Older	
	*M*	SD	*M*	SD
Age (years)^a^	23.0	1.4	73.3	4.1
Years of education^a^	14.5	1.2	19.4	3.2
Snellen letter acuity	10/11.1	1.5	10/14.1	2.5
Log contrast sensitivity^a,b^	1.69	0.13	1.52	0.22
Digit span forward	11.3	2.1	9.9	1.9
Digit span backward	8.9	1.7	7.2	1.6
Perceptual encoding manual^a^	88.8	17.8	70.7	18.3
Kaufman brief intelligence test	25.5	4.1	28.7	5.8

*^a^Differences between age groups were significant (*p* ≤ 0.05). ^b^Contrast sensitivity was measured using the Pelli Robson test (Pelli et al., 1988)*.

### Apparatus

Stimuli were presented on a Dell^®^ t3500 workstation using an Nvidia Quatro FX video card. The monitor was a Viewsonic P225 perfect flat CRT monitor set at a resolution of 1025 × 768 and operating a 120 Hz. Viewing distance was set at 80 cm and was controlled using a chin rest. The display was modulated by a Cambridge Research Bitts++ system running in Mono++ mode. This system allows for 16,384 distinct grayscale levels (14 bit precision). The monitor’s gamma was corrected to produce linear luminance output. The experiment was programmed in the Matlab (R2009a) environment using the Psychophysics Toolbox extension software (Brainard, 1997; Pelli, 1997). The experiment took place in a dark room. Participants responded using a standard keyboard.

### Stimuli

The stimuli were moving Gaussian filtered sine-wave gratings (Gabor patches). The Gabor patches had a spatial frequency of 1 cycle/°. Weber contrast (luminance − mean/mean) of the Gabor patch was set at one of three levels – 2.8, 22, or 92%. Grating contrast decreased from the center of the patch outward by a Gaussian envelope with standard deviation of 2°. Two different sizes were examined 0.7 of 5.0°. The three contrast levels and two sizes resulted in six unique conditions. The background luminance of the display was 36.6 cd/m^2^. On each trial the Gabor patch drifted to the right or left at a rate of 2°/s. The motion direction was determined randomly before each trial. On every trial the starting phase of the Gabor was randomized. The stimulus was presented within a square temporal window while the actual stimulus duration was manipulated on a trial-by-trial basis.

### Task and procedure

The task was to indicate if the motion direction of the Gabor patch was leftward or rightward. Participants were informed that the motion direction was determined randomly before each trial. If the participant could not judge the motion direction they were told to make their best possible judgment.

The experiment took place over 5 days. Days 1 and 5 were treated as pre- and post-testing days with days 2, 3, and 4 treated as training days. On each day the participant was presented with a random order of six blocks that represented all possible combinations of the three contrast levels (2.8, 22, and 91%) and two sizes (0.7 or 5°). On day 1, prior to experimental data collection, each participant completed an introduction program that presented 10 trials at maximum duration for each of the six blocks to introduce the stimuli, task, and procedure. At the beginning of each trial during the experiment the message “Press a key to start next trial” was displayed on screen. The participant initiated each trial by pressing any key on the keyboard causing the screen to go blank (set to the mean background luminance). After a delay of 500 ms a fixation point appears that flickered between white and black every 400 ms for 1.6 s. After which the display went blank for an additional 500 ms before the stimuli appeared. After the stimulus was presented the screen went blank and the participant entered their judgment by pressing the left or right arrow keys on the keyboard. The program waited for the response and produced a high tone if the response was correct or a low tone if the response was incorrect.

Each block consisted of 150 trials. After the 75th trial the program prompted the participant to take a short break. During this time the participant could not resume the experimental trials for at least 10 s. The participants were instructed to take as long as needed to avoid fatigue. At the end of the block the participant was prompted to inform the experimenter that a block was completed and to take a brief break. Each block took between 7 and 12 min to complete based on the time taken during the rest period and how fast the participant responded to each trial.

Thresholds were derived by manipulating the duration of the stimulus using two randomly interleaved staircases. A 2/1 staircase tracked the 71% point and a 4/1 estimated the 84% correct point. The two thresholds were averaged to produce a final estimated threshold of 77.5%. The range of possible durations was 500 ms (60 frames) to 16.6 ms (two frames). For each block the staircases were initialized at 500 ms. The step sizes were adjusted for both staircases for each reversal in the following order – 250 ms (30 frames), 125 ms (15 frames), 125 ms (15 frames), 46.6 ms (5 frames), 46.6 ms (5 frames), 25 ms (3 frames), 25 ms (3 frames), and 8.3 ms (1 frame). After the sixth reversal the step size remained at 8.3 ms (1 frame). The final threshold for both staircases was the average of reversal points excluding the first reversal. Each observer completed 900 trials each day for a total of 4500 trials over the entire experiment.

## Results

### Baseline performance

**Figure 1:**
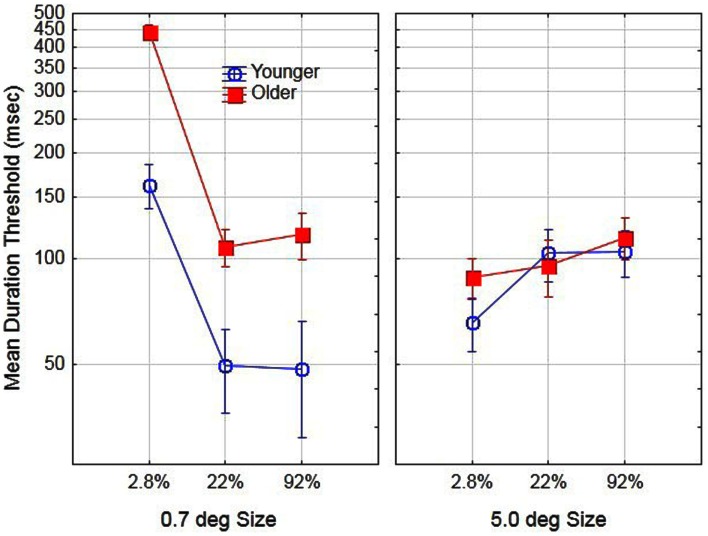
**Effects of stimulus size, contrast, and age on motion duration thresholds prior to training**. Error bars represent ±1 SE.

To examine age-related differences in performance prior to training, we conducted a two (age) by two (size) by three (contrast) analysis of variance (ANOVA) on day 1 (pre-training) using the log transformed thresholds for each subject. The main effect of aging was significant, *F*(1, 16) = 9.5, partial η^2^ = 0.37, *p* < 0.05. The mean duration threshold for older and younger observers was 161 and 81 ms, respectively. In addition, the main effects of size [*F*(1, 16) = 10.2, partial η^2^ = 0.38] and contrast [*F*(2, 32) = 48.5, partial η^2^ = 0.75] were significant, *p* < 0.05. The effects of these variables were mediated by age. Specifically, the age by size interaction [*F*(1, 16) = 31.9, partial η^2^ = 0.66] and age by contrast interaction [*F*(2, 32) = 4.3, partial η^2^ = 0.21] were significant, *p* < 0.05. Finally the age by size by contrast interaction was not significant, *F*(2, 32) < 1, *p* > 0.05 and for comparison purposes is shown in Figure 1. The general pattern of results is consistent with those reported by Tadin et al. (2003). For small size stimuli thresholds decreased with increased contrast whereas for large-size stimuli thresholds increased with increased contrast. This pattern of results occurred for both younger and older participants, with a much greater increase in thresholds for low-contrast/small stimuli for older as compared to younger participants. The present results, however, are not consistent with the results of Betts et al. (2005). According to their findings, older observers, as compared to younger observers had lower duration thresholds for large high-contrast stimuli. In the present study, we did not find any combination of size and contrast conditions that resulted in significantly lower duration thresholds for older as compared to younger observers.

### Training effects

**Figure 2:**
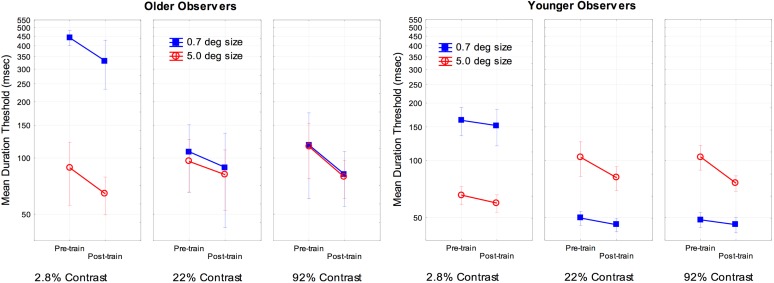
**Effects of training, size, contrast, and age on motion duration thresholds**. Error bars represent ±1 SE.

To examine the effects of training we conducted a two (age) by two (size) by three (contrast) by two (pre- and post-training) ANOVA on the log transformed thresholds for each subject. The main effect of age [*F*(1, 16) = 7.4, partial η^2^ = 0.31], training [*F*(1, 16) = 44.7, partial η^2^ = 0.73], and the interaction of age and training [*F*(1, 16) = 5.3, partial η^2^ = 0.25] were significant, *p* < 0.05. An analysis of the simple main effect of training indicated a significant effect for both older and younger observers, with a greater effect size of training occurring for older [*F*(1, 8) = 67.8, partial η^2^ = 0.89] as compared to younger subjects [*F*(1, 8) = 6.8, partial η^2^ = 0.46]. There were no other significant interactions with age and training (*F* values approximately 1). For comparison purposes the overall results are shown in Figure 2. As is shown in Figure 2, training resulted in improved performance for older subjects for all combinations of size and contrast examined, with a 23% improvement for the most difficult conditions (small-size and low-contrast).

### Spatial suppression

**Figure 3:**
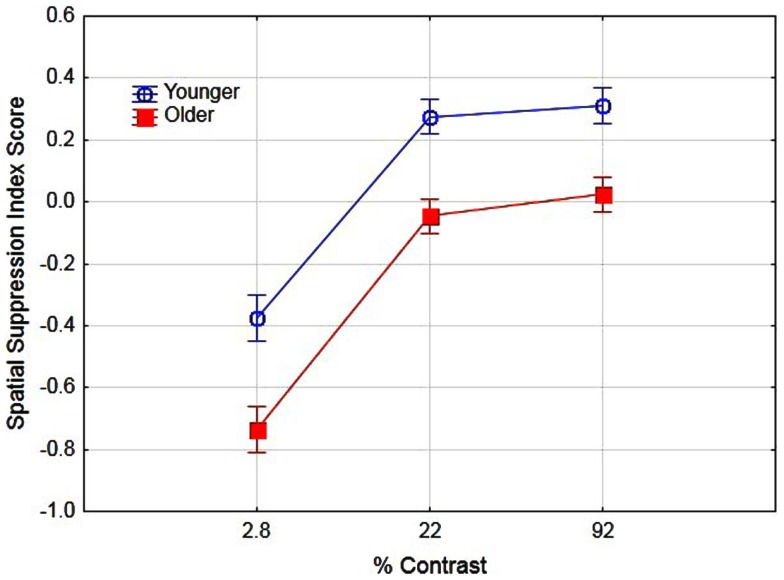
**Effects of contrast and age on spatial suppression index scores**. Error bars represent ±1 SE.

**Figure 4:**
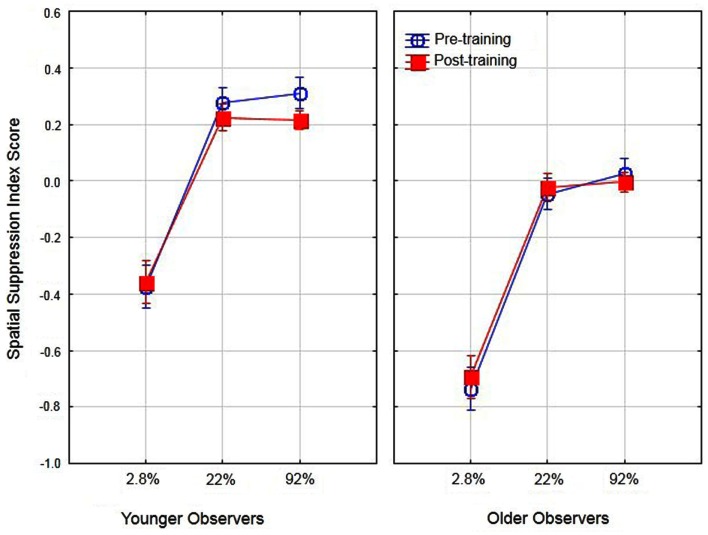
**Effects of training, contrast, and age on spatial suppression index scores**. Error bars represent ±1 SE.

Previous research has suggested that better performance for older observers in detecting large high-contrast motion targets is due to age-related differences in spatial suppression (Betts et al., 2005). In their research, they estimated spatial suppression by calculating a spatial suppression index: the log thresholds for large targets minus the log threshold for the smallest target at each level of contrast. The sign of these scores provide evidence of spatial suppression (positive scores) or spatial summation (negative scores). In the present study we conducted a similar analysis by deriving the log threshold for large (5.0°) targets minus the log threshold for the small target (0.7°) for each level of contrast. To examine the effects of aging on spatial suppression we conducted a two (age) by three (contrast level) ANOVA on spatial suppression index scores. The main effects of age [*F*(1, 16) = 31.1, partial η^2^ = 0.66] and contrast [*F*(2, 32) = 91.6, partial η^2^ = 0.85] were significant (*p* < 0.05). These results (see Figure 3) do provide evidence of reduced spatial suppression for older observers as compared to younger observers. However, the results indicate general age-related declines in spatial suppression (across all contrast levels) as opposed to declines exclusively for high-contrast targets. The interaction of age and contrast was not significant [*F*(2, 32) = 1.19]. These values were then calculated pre- and post-training to determine whether the use of PL training resulted in changes in spatial suppression and analyzed in a two (age) by two (training) by three (contrast) ANOVA. The overall results are shown in Figure 4. The main effect of age was significant, *F*(1, 16) = 39.1, partial η^2^ = 0.70, *p* < 0.05 indicating lower overall spatial suppression scores for older (mean = −0.24) as compared to younger (mean = 0.04) subjects. In addition, the main effect of contrast was significant, *F*(2, 32) = 121.7, partial η^2^ = 0.88, *p* < 0.05 indicating lower suppression index scores for low as compared to high-contrast stimuli. The main effect of training was not significant nor did training interact with age or contrast (*F* values less than 1.3). These results suggest that the effects of training did not result in changes in spatial suppression.

## Discussion

The results of the present study indicate several important findings concerning motion processing and aging. First, consistent with previous research (Gilmore et al., 1992; Atchley and Andersen, 1998; Betts et al., 2005, 2009, 2012; Bennett et al., 2007) we found that older observers, as compared to younger observers, have increased motion discrimination thresholds. In addition, the ability to determine the direction of motion decreased with a decrease in contrast of the motion stimulus especially for small targets. Previous research (Betts et al., 2005) has reported improved motion thresholds for older as compared to younger observers for large high-contrast stimuli – a result interpreted to be associated declines in inhibition and changes in spatial suppression. In the present study we did not find this pattern of results. We did, however, find evidence of general age-related declines in spatial suppression across all contrast levels examined. So what might account for the failure to replicate this interesting finding regarding age, spatial suppression, and high-contrast stimuli? We currently do not have an explanation to account for these differences. There are some interesting differences between the two studies regarding the older subjects (e.g., differences in baseline performance; the degree to which subjects might be considered high cognitive functioning) that might contribute to the differences obtained in spatial suppression. Clearly future research is needed to understand the different age effects obtained in the two studies.

The results of the present study also indicate an effect of training for older observers. We found an overall improvement in performance for older individuals as compared to younger individuals. In addition, training resulted in improvement for older observers for all combinations of contrast and size examined, with the greatest magnitude of improvement occurring for the small low-contrast target condition. Younger subjects also showed improvement due to training, but the effect size of training was lower than that observed with older subjects. These results provide further evidence that PL training is a useful procedure for improving vision among older individuals. Indeed, several of the older observers at the beginning of the study commented that they were unable to see the small low-contrast stimuli. However, following training these same individuals commented that they could easily see targets from these stimuli conditions. These results indicate a significant degree of improvement in motion thresholds for older observers as a result of 3 days of training.

The results regarding the spatial suppression index indicate that both older and younger observers have increased suppression with increased contrast, a finding consistent with previous research (Tadin et al., 2003) that showed greater suppression for large high-contrast stimuli. However, we did not find any evidence that training resulted in changes in suppression for either age group. These findings may be limited to the conditions (size and contrast) examined in the present study. In addition, it is possible that additional training beyond 3 days may be required to result in changes in spatial suppression. An important issue for future research will be to examine this possibility with a greater range of stimulus conditions.

In summary, the results of the present study provide further evidence of the utility of PL methods to improve vision among older individuals. The results suggest that training was effective for small low-contrast stimuli – conditions that were the most difficult for older observers prior to training. In addition, although we found evidence of general age-related differences in spatial suppression, we did not find evidence that training resulted in changes in suppression for either younger or older participants. Previous research (Bower and Andersen, 2012) has found evidence of changes in internal noise for older observers as a result of training with motion stimuli and has suggested that this finding might be due to changes in inhibition. Given that the present study did not find evidence of changes in inhibition, it suggests that other factors may be important in changing efficiency in motion processing.

## Conflict of Interest Statement

The authors declare that the research was conducted in the absence of any commercial or financial relationships that could be construed as a potential conflict of interest.
